# Improved surgical procedure of primary constrained total knee arthroplasty which enables use of the femoral diaphyseal straight extension stem

**DOI:** 10.1186/s12891-022-05367-w

**Published:** 2022-05-02

**Authors:** Shinya Kawahara, Taro Mawatari, Gen Matsui, Satoshi Hamai, Yukio Akasaki, Hidetoshi Tsushima, Yasuharu Nakashima

**Affiliations:** 1 Department of Orthopaedic Surgery, Graduate School of Medical Sciences, Kyushu University, 3-1-1 Maidashi, Higashi-ku, Fukuoka, 812-8582 Japan; 2 Department of Orthopaedic Surgery, Hamanomachi Hospital, 3-3-1 Nagahama, Chuo-ku, Fukuoka, 810-8539 Japan

**Keywords:** Total knee arthroplasty, Constrained condylar knee, Straight stem, Osteoarthritis

## Abstract

**Background:**

In performing primary constrained total knee arthroplasties (TKA) to imbalanced knees, the offset stem is sometimes compelled to use, although this is associated with surgical difficulties. We developed a modified procedure which might be able to fit the anteroposterior (AP) and mediolateral (ML) position of the femoral component simultaneously with the straight stem. Purposes of this study were to evaluate usefulness of the modified procedure both in computer simulations and actual surgeries.

**Methods:**

We included 32 knees that had undergone primary TKA using constrained implants because of the coronal imbalance. In the component-first procedure, the distal femur was prepared to fit the AP and ML position of the femoral component simultaneously at first, as in primary TKA. Finally, the stem hole is created based on the femoral component position (the component-first procedure). The femoral component and extension stem were simulated using the three-dimensional planning software (ZedKnee) following the component-first procedure. We investigated the suitability of the straight stem through computer simulation and evaluation of actual surgeries. Clinical and radiographical outcomes were also evaluated at the latest follow-up.

**Results:**

The component-first procedure enabled the AP and ML position of the femoral component to be fitted simultaneously with the straight stem in simulations and actual surgeries in all cases. The stem diameter was not significantly different between simulations and actual surgeries (13.9 and 13.7 mm on average, respectively, *p* = 0.479) and almost similar from intraclass correlation coefficient analysis (kappa value 0.790). Clinical and radiographical outcomes were almost similar to primary TKA cases and there was no case of component loosening, cortical bone hypertrophy around the stem and stem-tip pain.

**Conclusions:**

Our improved surgical procedure may facilitate use of the constrained implant for more cases of primary TKA in imbalanced knees without the usual surgical difficulties.

**Trial registration:**

Retrospectively registered.

## Background

Ongoing ligament instability is a common reason for early revision of total knee arthroplasty (TKA) [1–4]; however, severe instability of the knee in the coronal plane is difficult to correct using a cruciate-retaining (CR) or posterior-stabilized (PS) implant [5]. Typically used in revision TKA, constrained condylar knee (CCK) implants are sometimes used in primary TKA to improve stability in the coronal plane in patients with severe varus/valgus deformities, capsule-ligament instability, or rheumatoid arthritis [5]. These implants include modular cemented or uncemented extension stems which reduce the risk of mechanical loosening by transferring a portion of the load to the intramedullary canal [5, 6]. In our institution, the diaphyseal extension stem have been preferably used for fear of the implant removal trouble in case of revision surgeries with stem cementing.

**Fig. 1:**
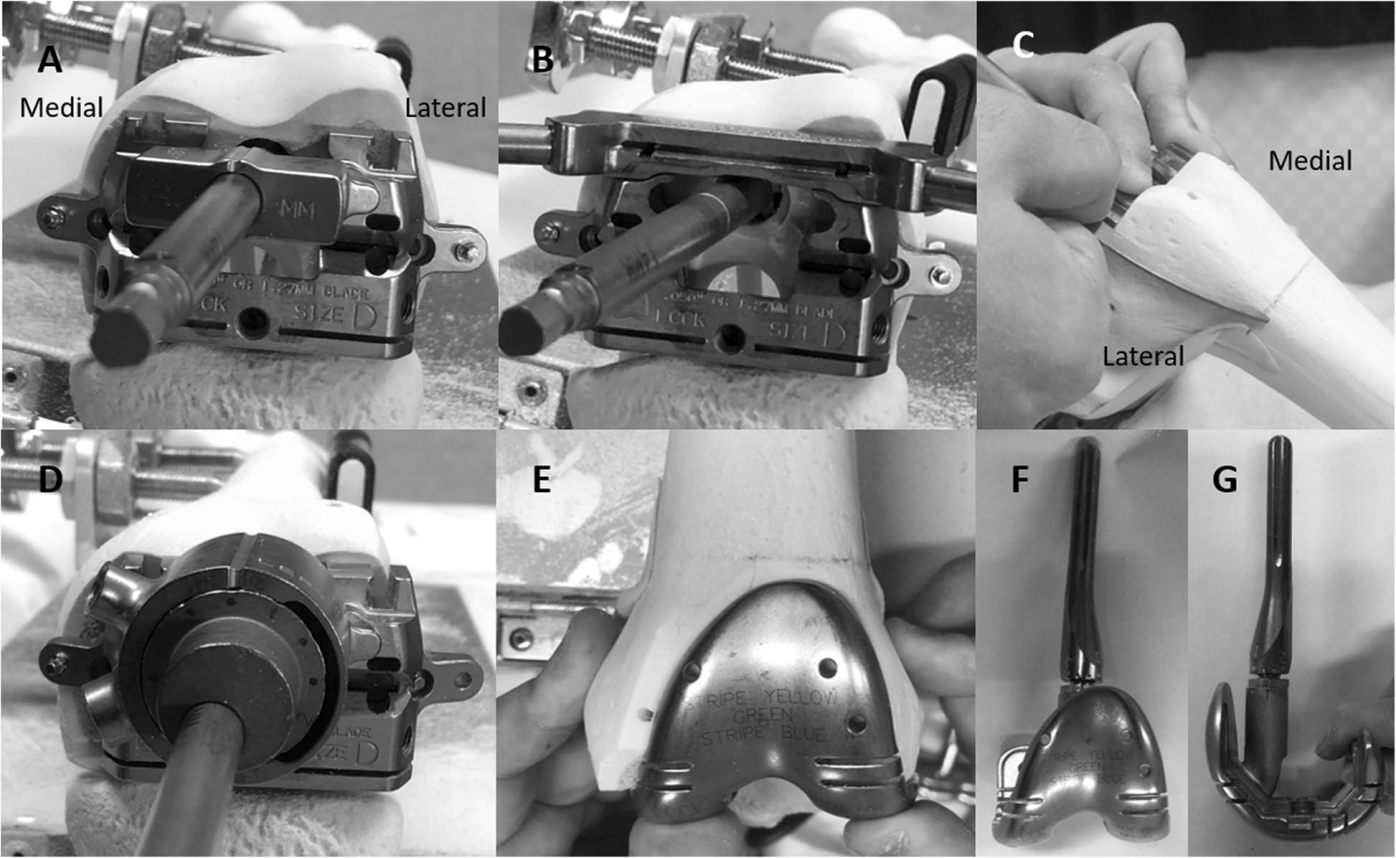
Stem-first procedure. **A** ML and **B** AP placement with the straight stem. **C** AP position is adjusted to avoid anterior notching by using the offset stem. **D**, **E** ML position is shifted by AP adjustment. **F**, **G** Femoral trial component with the offset stem

**Fig. 2:**
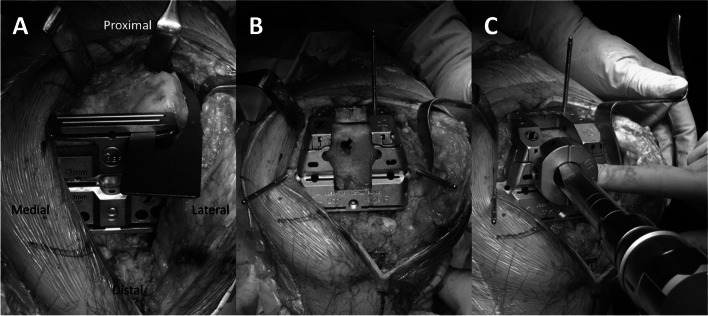
Component-first procedure. **A** Anterior resection is performed parallel to the SEA to avoid anterior notching. **B** ML position is adjusted based on the anterior and distal resection surface. **C** The stem hole is created based on the position

**Fig. 3:**
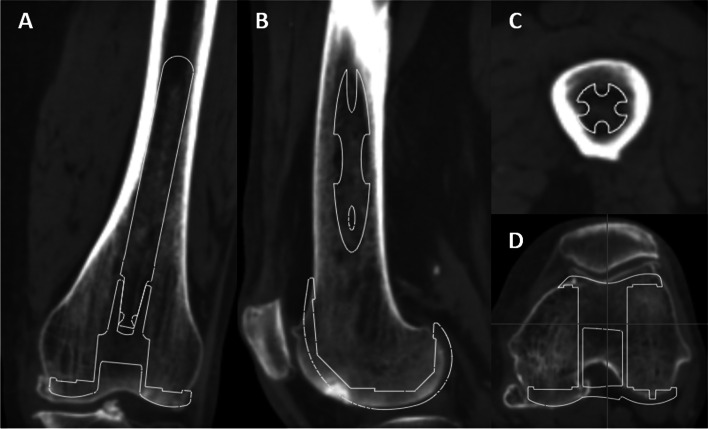
Three-dimensional templating of the femoral component and the straight extension stem. **A** Coronal and **B** sagittal views. **C**, **D** Axial views on the stem tip level and the component level, respectively

The recommended surgical approach for primary constrained TKA involving distal femoral components is to first decide the position and diameter of the extension stem. However, the anteroposterior (AP) and mediolateral (ML) placement of the femoral component simultaneously with the straight stem is often challenging (Fig. 1A and B), and the offset stem is used to adjust AP and ML in some cases (Fig. 1C-G; this is defined as the “stem-first procedure” in the present study), which requires considerable technical expertise. Therefore, we routinely used the following surgical procedures: (1) Resection of the distal femur with reference to the intramedullary rod, with anterior resection performed parallel to the surgical epicondylar axis (SEA; the axis connecting the tip of the lateral epicondyle and the medial epicondylar sulcus [7–9]) to avoid anterior notching (Fig. 2A); (2) Adjustment of the ML position of the femoral component to minimize overhang and underhang to the maximum extent possible based on the anterior resection, as for primary TKA (Fig. 2B); (3) Creation of the stem hole based on the position of the femoral component position (Fig. 2C; this surgical procedure is defined as the “component-first procedure” in the present study). This procedure needs no special instrument and can be performed by all surgeons.

The component-first procedure simultaneously avoids anterior notching and compensates for the ML balance of the femoral component; however, it is not clear whether the use of an intramedullary-occupied straight stem is suitable, even in the procedures that we routinely perform.

The present study aimed (1) to investigate whether adjustment of the femoral component position by three-dimensional (3D) templating during primary CCK TKA ensured the appropriate position and diameter of the femoral straight extension stem and (2) to evaluate the suitability of a straight extension stem in primary TKA using a CCK implant with the component-first procedure, and (3) to evaluate short-term clinical and radiographical outcomes.

## Methods

We recruited all consecutive patients with varus osteoarthritis (OA), valgus OA, or rheumatoid arthritis (RA) with medial instability or osteoarthritis after high tibial osteotomy (HTO) who underwent primary TKA using a CCK implant; specifically, the NexGen Legacy Constrained Condylar Knee (LCCK; Zimmer Biomet Inc., Warsaw, IN, USA); between April 2014 and August 2018. CCK implants were prepared in addition to regular TKA implants when surgeons judged the ML instability in the preoperative manual examination. Indications for primary CCK TKA were finally determined based on the perioperative gap measurements with insufficient coronal balancing and > 5° laxity, or a flexion/extension gap mismatch of > 3 mm and < 2 cm [10–13]. There was no case excluded due to the femoral bowing. All patients provided informed consent and the study and all its protocols were approved by the local institutional review board of the authors’ affiliated institutions (Hamanomachi Hospital, No. 2018–09). All methods were performed in accordance with the relevant guidelines and regulations.

Preoperative transverse computed tomography (CT) images (Aquilion ONE; Canon Medical Systems Corporation Japan, Tochigi, Japan) were obtained at levels from the hip to ankle joints at 1.25-mm intervals and 1.25 mm thickness with a field of view of 400 and pitch of 1.375. Patients were placed in the supine position for CT examination, and the affected knee was naturally extended, monitoring for any feeling of internal or external rotation. Images were acquired in Digital Imaging and Communications in Medicine format (DICOM) from the software of the CT scanner.

### Three-dimensional templating of the femoral component and the straight extension stem

We imported DICOM data sets into 3D pre-operative planning software (ZedKnee; Lexi, Tokyo, Japan), and 3D femoral bone models were reconstructed using the software with the 3D coordinate system embedded into them. The femoral mechanical axis was defined as the line connecting the center of the femoral head and the midpoint of the SEA. The coronal plane was defined as the plane of the femoral mechanical axis and the SEA. The anatomical axis of the distal femur was determined automatically using the software.

The femoral component was templated by referring to the alignments described below. The coronal alignment was set at 6° valgus relative to the anatomical axis of the distal femur considering the specific valgus angle of the extension stem of the CCK implant. Sagittal alignment was set parallel to the anatomical axis of the distal femur to avoid notching [14, 15], and rotational alignment was parallel to the SEA. The size of the femoral component was chosen as the best match for the AP dimension of the native femoral lateral condyle [16]. The ML position was set to minimize overhang and underhang as much as possible [17].

**Fig. 4:**
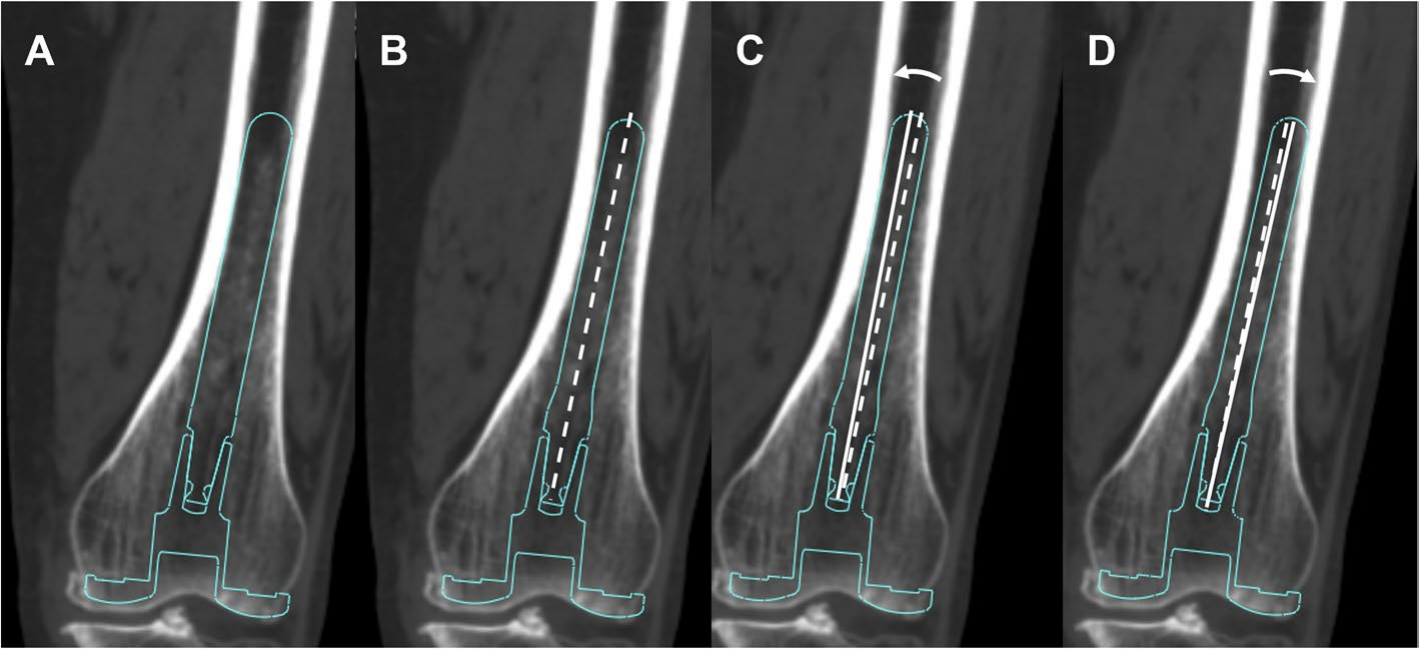
**A** Three-dimensional templating of the straight extension stem (maximum diameter). **B** The extension stem down-sized by 1 or 2 mm is aligned (broken line: extension stem axis) and deviated at maximum to touch **C** medial and **D** lateral side of the intramedullary wall, respectively (solid lines: deviated extension stem axes)

To investigate the suitability of inserting a straight extension stem into the intramedullary canal of the distal femur, we used a 100-mm extension stem as per the manufacturer’s recommendation. The extension stem was set to avoid invasion of the intramedullary wall of the femoral cortex (Fig. 3). First, whether or not the straight extension stem could be used was investigated. Then, we measured the maximum diameter of the straight extension stem in cases where it was able to be used. Next, we measured the acceptable varus-valgus and extension-flexion angle deviations of the extension stem when the stem diameter was down-sized by 1 or 2 mm, respectively, relative to the maximum diameter (Fig. 4).

### Actual primary TKA using a CCK implant

Primary TKA was performed using a CCK implant in these all knees. The distal femur was prepared so as to reproduce the simulation described above using the intramedullary rod. Whether or not the straight extension stem could be used was investigated again. We should evaluate the reproducibility of the 3D templating to the actual surgery and the stem diameter in actual surgery was compared to that in 3D templating.

### Short-term evaluation of clinical and radiographical outcomes

All patients were followed regularly after surgery. Range of motion (ROM), radiographs and The Knee Society Score 2011 [18], were assessed at the latest follow-up. The coronal alignments of whole-leg, femoral and tibial components are measured and the existence of component loosening and cortical bone hypertrophy around the stem were evaluated.

### Statistical analysis

Maximum diameter of the straight extension stem was compared between computer simulation and actual surgery using Wilcoxon signed-rank sum test. In addition, its rate of concordance between each measurement was evaluated as intraclass correlation coefficient (ICC). All 3D templating and measurements were carried out twice by one examiner with an interval of at least 1 month and once by another examiner on the study group. Intra- and inter-observer differences in templated femoral component size and maximum stem diameter were evaluated as ICC. JMP 14.3 (SAS Institute Inc., Cary, NC, USA) was used to analyze the data. Significance was set at a *p*-value of < 0.05.

## Results

In total, we recruited 24 patients (32 knees) for the present study. Patient characteristics are detailed in Table 1. All patients were Japanese. The component-first procedure compensated the appropriate AP and ML positions of the femoral component without anterior notching or anterior flange spacing both in computer simulations and actual surgeries in all cases. The femoral component size used in actual surgeries completely matched that in computer simulations. The straight extension stem could be templated based on our computer simulations and were able to be used in actual surgeries in all cases. The stem diameter was not significantly different between simulations and actual surgeries from Wilcoxon signed-rank sum test (*p* = 0.479) and almost similar from ICC analysis (13.9 and 13.7 mm on average, respectively, kappa value 0.790; Table 2). The acceptable varus-valgus and extension-flexion angle deviations of the extension stem with down-sizing of the stem diameter in simulations are presented in Table 3. When the stem diameter was down-sized by 2 mm, the varus-valgus angle deviation of the extension stem was almost 1° on average. Clinical and radiographical outcomes were described in Table 4. Though all cases were followed in a short period (duration of follow-up: 3.9 ± 1.3 years), they were almost similar to primary TKA cases described in the previous work of Matsuda et al. [19] and there was no case of component loosening, cortical bone hypertrophy around the stem and stem-tip pain.

**Table 1:** Patient details

		**24 Patients, 32 Knees**	
Age (yr)		76.0 ± 7.3	
Gender		M 5 F 19	
Side (Rt / Lt)		Rt 14 Lt 18	
Height (cm)		151.0 ± 6.7	
Body weight (kg)		56.9 ± 9.4	
Body mass index (BMI)		25.0 ± 4.2	
Preoperative alignment		Varus 29 knees (> 15°: 24, ≤ 15°: 5)	
		Valgus 3 knees (all > 10°)	
Preoperative diagnosis and knee alignment			
Diagnosis	varus / valgus (knees)		knee alignment
OA	varus 25		20.0 ± 7.2°
	valgus 1		12.5°
RA	varus 3		21.7 ± 8.1°
	valgus 1		10.3°
OA after HTO	varus 1		10.2°
	valgus 1		10.2°

**Table 2:** Number of knees each stem diameter is used

Stem diameter (mm)	3D templating (knees)	Surgery (knees)
10	1 (3.1%)	
11	2 (6.3%)	3 (9.4%)
12	5 (15.6%)	6 (18.8%)
13	4 (12.5%)	3 (9.4%)
14	5 (15.6%)	8 (25.0%)
15	9 (28.1%)	9 (28.1%)
16	6 (18.8%)	3 (9.4%)
Mean ± Standard deviation	13.9 ± 1.7 mm	13.7 ± 1.5 mm
Wilcoxon signed-rank sum test	*p* = 0.479	
Intraclass correlation coefficient	Kappa value 0.790

**Table 3:** Varus-valgus and extension-flexion angle deviations of the extension stem with down-sizing of the stem diameter

	1 mm down-sizing	2 mm down-sizing
Varus	0.6 ± 0.4°	0.9 ± 0.4°
Valgus	0.7 ± 0.3°	1.0 ± 0.3°
Varus-valgus	1.3 ± 0.6°	1.9 ± 0.7°
Extension	0.9 ± 0.4°	1.3 ± 0.5°
Flexion	0.8 ± 0.4°	1.1 ± 0.5°
Extension-flexion	1.7 ± 0.7°	2.4 ± 1.0°

All values are given as the mean and standard deviation

**Table 4:** Clinical and radiographical outcomes

Duration of follow-up	3.9 ± 1.3 years	
Knee extension angle^a^	-2.9 ± 4.0°	
Knee flexion angle	122.9 ± 9.4°	
Coronal alignment		
Whole-leg	1.2 ± 1.0° varus	
Femoral component	0.9 ± 1.0° varus	
Tibial component	0.3 ± 0.5° varus	
The Knee Society Score 2011		
Subscale (full marks)	This study	Matsuda et al. [15]
Symptom score (25)	20 ± 5 (82%)	19 ± 6 (74%)
Satisfaction score (40)	27 ± 8 (68%)	23 ± 8 (59%)
Expectation score (15)	12 ± 4 (76%)	10 ± 3 (64%)
Functional activities score (100)	73 ± 18 (73%)	53 ± 23 (53%)

^a^Extension and flexion angles were described as positive and negative values, respectively

The intra- and interobserver reproducibilities of 3D templating of the femoral component and the straight extension stem were almost excellent (ICC > 0.9 for all templating parameters which indicates that all the 3D templating had good reliabilities).

## Discussion

The most important finding of the study is that the straight extension stem is suitable for use in TKA as demonstrated by both computer simulation and actual surgery using the component-first procedure. It is often difficult to simultaneously fit the AP and ML position of the femoral component to avoid anterior notching and compensate for ML balance with the straight stem using the stem-first procedure (which is generally recommended as the routine procedure for CCK TKA). Examples of the stem-first procedure are shown in Fig. 1. In the case shown in Fig. 1B, surgeons take care of anterior notching and the offset stem would be used. The offset length of the NexGen LCCK implant is 4 mm, meaning that they are forced to move the femoral component $\sqrt{{4}^{2}-{x}^{2}}$ mm mediolaterally when the AP position is adjusted by *x* mm, which can cause difficulties (Fig. 1C-E). In this respect, the component-first procedure would facilitate use of the CCK implant for primary TKA in cases of coronal imbalance without these difficulties.

During distal femur resection, it is possible that slight angular errors could occur by using the intramedullary rod or actual bone resection, which may result in impingement of the straight extension stem. Haruta et al. described the maximum deviation of the intramedullary rod to be almost 1° in the coronal plane [20], in line with the results of the present study. Therefore, a slight angular error in the surgical procedure could be compensated by slight down-sizing of the extension stem diameter.

The optimal level of constraint in knees with ligamentous laxity has not been clearly defined. Indications for primary CCK TKA are described as end-stage varus or valgus knee osteoarthritis with insufficient coronal balancing and > 5° laxity, or a flexion/extension gap mismatch of > 3 mm and < 2 cm [10–13]. However, the decision to perform primary CCK TKA tends to be made irrespective of surgeons’ concerns regarding mid- and long-term clinical outcomes and the difficulties associated with the surgical procedure. In terms of clinical outcomes, the 10-year survival rate of primary CCK TKA has been reported to be 96%–100%, which is not considerably inferior to primary TKA [6, 13, 21, 22]. In addition, cortical bone hypertrophy around the stem and stem-tip pain are rarely identified [6, 13, 21–24]. In our series, though a short-term follow-up, clinical and radiographical outcomes were almost similar to primary TKA cases described in the previous work of Matsuda et al. [19] and there was no case of component loosening, cortical bone hypertrophy around the stem and stem-tip pain. In terms of surgical difficulties, the primary difficulty is associated with the femoral procedure described above, whereas the tibial procedure is unlikely to cause difficulties except in cases where tibial deformities such as postoperative closed-wedge HTO are present. The component-first procedure presented here allows the CCK implant to be used with relative ease. Therefore, primary CCK TKA may be a more acceptable approach for older patients with severe coronal malalignment and imbalance.

There are some limitations to this study which should be acknowledged. Firstly, the sample size was small. Primary CCK TKA is rarely performed and most were small cases [13, 25]. Nevertheless, we believe that the cases included in this study are sufficient for the present investigation because the straight extension stem was able to be used in both computer simulations and actual surgeries in all cases. Secondly, the study population was limited to Japanese subjects. There are several anatomical differences between Japanese and Caucasian individuals [26, 27], and body size differs between races. Therefore, the results should be interpreted with caution. Thirdly, our study did not examine the mid- and long-term outcomes of primary CCK TKA using the component-first procedure. Therefore, the cases included in this study should be carefully followed in future. Finally, the improved surgical procedure only in case of using the specific 100-mm femoral diaphyseal straight extension stem has been described in this study. Cementless diaphyseal stems and cemented metaphyseal stems have different fixation concepts and specific advantages and disadvantages with each. On the other hand, constrained TKA without extension stems has also been reported to have good mid-term outcomes in several studies [28, 29]. There has been no prospective research which surgical technique is more preferable and further studies should be performed in the future.

## Conclusions

We demonstrate through the results of actual surgery and computer simulation that our component-first procedure enables surgeons to fit the AP and ML position of the femoral component simultaneously to avoid anterior notching and compensate for ML balance using a straight stem. This surgical procedure may enable surgeons to use the CCK implant in more cases of primary TKA in imbalanced knees with minimal difficulty.

## Data Availability

All data generated or analysed during this study are included in this published article.

## Abbreviations

TKA: Total knee arthroplasty
AP: Anteroposterior
ML: Mediolateral
OA: Osteoarthritis
CCK: Constrained condylar knee
CR: Cruciate-retaining
PS: Posterior-stabilized
SEA: Surgical epicondylar axis
3D: Three-dimensional
RA: Rheumatoid arthritis
HTO: High tibial osteotomy
CT: Computed tomography
DICOM: Digital Imaging and Communications in Medicine format
ROM: Range of motion
ICC: Intraclass correlation coefficient
